# A novel pathological mutant reveals the role of torsional flexibility in the serpin breach in adoption of an aggregation‐prone intermediate

**DOI:** 10.1111/febs.17121

**Published:** 2024-03-24

**Authors:** Kamila Kamuda, Riccardo Ronzoni, Avik Majumdar, Fiona H. X. Guan, James A. Irving, David A. Lomas

**Affiliations:** ^1^ Division of Medicine, UCL Respiratory, Rayne Institute University College London UK; ^2^ Institute of Structural and Molecular Biology, Birkbeck College University College London UK; ^3^ AW Morrow Gastroenterology and Liver Centre Royal Prince Alfred Hospital Sydney Australia; ^4^ Victorian Liver Transplant Unit Austin Health Melbourne Australia; ^5^ The University of Melbourne Melbourne Australia

**Keywords:** alpha‐1‐antitrypsin, alpha‐1‐antitrypsin deficiency, conformational disease, protein folding, serpin

## Abstract

Mutants of alpha‐1‐antitrypsin cause the protein to self‐associate and form ordered aggregates (‘polymers’) that are retained within hepatocytes, resulting in a predisposition to the development of liver disease. The associated reduction in secretion, and for some mutants, impairment of function, leads to a failure to protect lung tissue against proteases released during the inflammatory response and an increased risk of emphysema. We report here a novel deficiency mutation (Gly192Cys), that we name the Sydney variant, identified in a patient in heterozygosity with the Z allele (Glu342Lys). Cellular analysis revealed that the novel variant was mostly retained as insoluble polymers within the endoplasmic reticulum. The basis for this behaviour was investigated using biophysical and structural techniques. The variant showed a 40% reduction in inhibitory activity and a reduced stability as assessed by thermal unfolding experiments. Polymerisation involves adoption of an aggregation‐prone intermediate and paradoxically the energy barrier for transition to this state was increased by 16% for the Gly192Cys variant with respect to the wild‐type protein. However, with activation to the intermediate state, polymerisation occurred at a 3.8‐fold faster rate overall. X‐ray crystallography provided two crystal structures of the Gly192Cys variant, revealing perturbation within the ‘breach’ region with Cys192 in two different orientations: in one structure it faces towards the hydrophobic core while in the second it is solvent‐exposed. This orientational heterogeneity was confirmed by PEGylation. These data show the critical role of the torsional freedom imparted by Gly192 in inhibitory activity and stability against polymerisation.

## Graphical abstract

Here, we characterised a novel pathological variant of alpha‐1‐antitrypsin with a single substitution of glycine to cysteine at position 192. This mutation is positioned within the breach region, which is crucial for inhibitory function of the protein. Glycine substitution leads to reduced flexibility within the breach region, alters protein activity and leads to alpha‐1‐antitrypsin polymer accumulation within the cell.

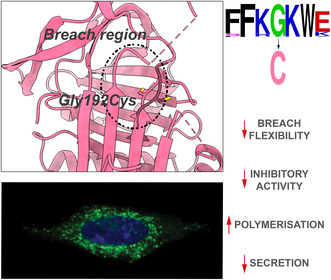

**Abbreviations**
5k PEG: methoxypolyethylene glycol maleimide
AAT: alpha‐1‐antitrypsin
AATD: alpha‐1‐antitrypsin deficiency
CD: far‐ultraviolet circular dichroism
COSM: centre of spectral mass
DTT: dithiothreitol
*E*_a_
: apparent activation energy
ER: endoplasmic reticulum
FRET: fluorescence resonance energy transfer
IF: immunofluorescence
*K*_SV_
: quenching constant
M*: polymerisation‐prone intermediate
RCL: reactive centre loop
SD: standard deviation
SEM: standard error of the mean
*T*_m_
: midpoint temperature of change

## Introduction

Newly synthesised proteins adopt a native fold required for proper function. In the event of misfolding in the cytoplasm or endoplasmic reticulum (ER), a polypeptide chain is directed along a degradative pathway. Inherited or acquired genetic mutations can have profound consequences for a protein's ability to fold, resulting in accumulation of aggregates. These lead to conformational diseases which encompass a diverse group of disorders across all tissues of the body [1]. Among these, the serpinopathies are characterised by the formation of self‐assembled, functionally inactive polymers of members of the serpin superfamily of proteinase inhibitors, predominantly accumulating within the lumen of the ER [2]. The chronically overloaded ER undergoes morphological distortion and dysfunction that is associated with disease.

**Fig. 1:**
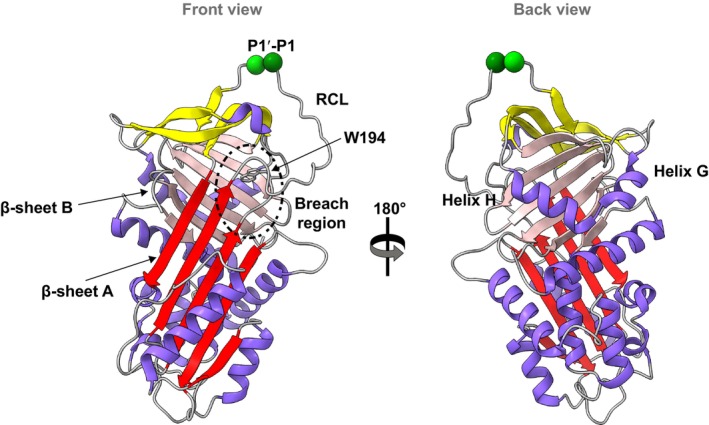
The native AAT structure. A canonical ‘front’ and ‘back’ view of AAT (PDB: 1QLP) [27] highlighting the functionally relevant breach region, which initiates insertion of the reaction centre loop (RCL) into β‐sheet A. Structures were generated using chimerax [56].

Alpha‐1‐antitrypsin (AAT) is a 52 kDa, 394 amino acid glycoprotein that represents the archetypal member of the serpin superfamily. It is predominantly synthesised within the liver and then secreted into the circulation where it carries out its function as a proteinase inhibitor, mainly inhibiting neutrophil elastase [3]. The structure of AAT is based around a central β‐sheet A and a 20 amino acid reactive centre loop (RCL) [4]. Many mutations of AAT have been described to destabilise the native protein allowing it to form ordered polymers that are retained as PAS‐positive, diastase‐resistant inclusions within the ER of hepatocytes [5]. The retention of these polymers is associated with cirrhosis and hepatocellular carcinoma while the reduced AAT secretion predisposes to early‐onset emphysema; these conditions represent the characteristic pathology associated with alpha‐1‐antitrypsin deficiency (AATD). The best‐characterised of the deficiency variants associated with AATD is the Z allele (Glu342Lys) [6], but different mutations including Siiyama (Ser53Phe) [7], Mmalton (Δ52) [8] and King's (His334Asp) [9] similarly form polymers that are retained within hepatocytes and predispose to liver disease. These and other naturally occurring mutants have played a fundamental role in advancing understanding of the structural determinants of stability, function and misfolding of serpins [10]. During inhibition of a target protease [11, 12] and likely during polymerisation [6], a serpin undergoes a conformational rearrangement in which β‐sheet A accommodates the RCL as a newly formed sixth strand. A key region involved in the initiation of this structural transition is the breach. The breach region is located in the vicinity of the top of the strands 3 and 5 of β‐sheet A, as well as strands 2, 3 and 4 of β‐sheet B [13] and serves as a primordial point of insertion of the RCL (Fig. 1).

We report here a novel mutant of AAT identified in an individual from Sydney, Australia. Characterisation of this variant in cellular models of disease and using structural studies showed that Cys192 perturbs the structure of the breach region of the protein to form the intracellular polymers that are associated with liver disease in AATD. The effect of this mutation highlights the dual role played by the breach in both inhibitory activity and polymerisation. Interestingly, in comparison with other mutants that we have characterised, this variant acts by destabilising the polymerisation intermediate.

## Results

### Clinical details

A 32‐year‐old male was identified at the Royal Prince Alfred Hospital in Sydney with abnormal liver enzymes; ALT, AST and gamma GT were elevated at 67 U·L^−1^, 43 U·L^−1^ and 50 U·L^−1^ respectively. Alkaline phosphatase and serum bilirubin were in the normal range and synthetic liver function was intact. There was no evidence of autoimmune and viral causes of liver disease. Liver stiffness measurement with Fibroscan® (Echosens, Paris, France) was 5 kPa in the non‐fasting state with a controlled attenuation parameter score (CAP) of 260. ΑΑΤ levels were low in a range between 0.36 and 0.54 g·L^−1^ (normal range 1.5–3.5 g·L^−1^), approximately in the range seen with individuals with a ZZ genotype [14]. Phenotyping revealed the presence of the Z allele (Glu342Lys) in heterozygosity with a second, unknown allele. Genotyping of the AAT (*SERPINA1)* gene identified a novel mutation c.646G>T transversion that results in the substitution of glycine to cysteine at position 192 (Gly192Cys). This new protein variant was named ΑΑΤ Sydney (here referred to as Gly192Cys) after the name of the city in which it was identified. The circulating concentration of AAT was below that seen with SZ heterozygotes and so this variant could be considered as severely deficient [15]. Lung function tests on this patient showed an FEV_1_ of 3.21 L (76% of predicted) and an FVC of 4.28 L (83% predicted; ratio 75% predicted). The FEV_1_ increased to 3.83 L after 400 μg of inhaled salbutamol (19% increase). Static lung volumes were normal and the corrected Kco was 111% of predicted.

### Gly192Cys AAT accumulates as polymers within the cell

**Fig. 2:**
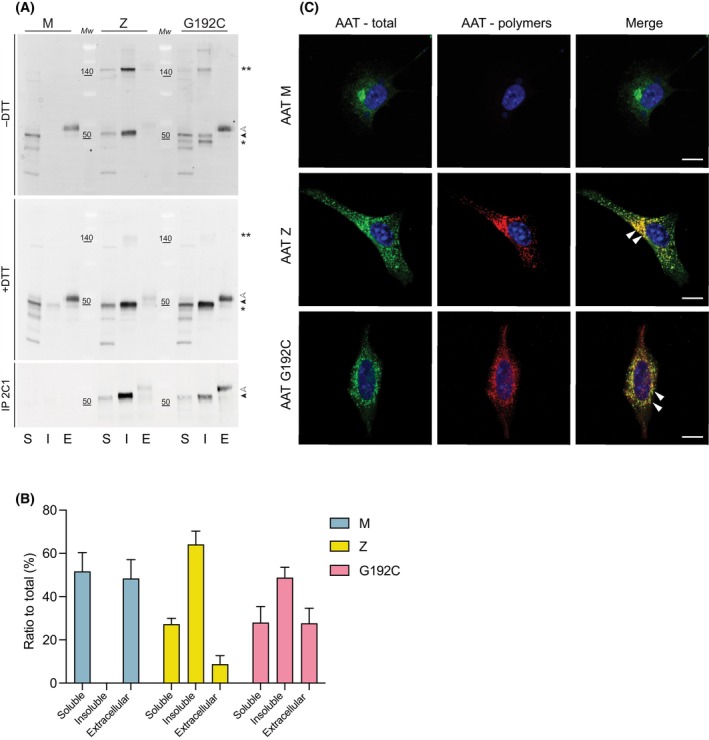
Gly192Cys AAT accumulates in cells as polymers in the soluble and insoluble intracellular fractions. (A) Hepa 1.6 cells were transfected as indicated and lysed in 1% v/v NP‐40 buffer 48 h after transfection. The soluble (S), insoluble (I) and extracellular (E) fractions were separated by 4–12% w/v acrylamide SDS/PAGE in non‐reducing (*top panel*) and reducing (*middle panel*) conditions and the proteins visualised by immunoblotting for total AAT with a polyclonal anti‐human AAT (Dako). The fractions were also immunoprecipitated with the anti‐polymer 2C1 mAb, eluted with an SDS‐based buffer and analysed by 4–12% w/v acrylamide SDS/PAGE followed by immunoblotting (*bottom panel*). White and black arrowheads indicate the mature and immature glycosylated forms of AAT, respectively. Single and double asterisks represent the intrachain form and interchain disulphide dimer, respectively. Mw indicates the molecular weight protein markers. The panel is representative of three independent experiments. (B) The percentage of intracellular and secreted AAT was calculated by densitometric quantification using imagej software [57]. The graph shows mean ± standard error of the mean (± SEM, *n* = 3). (C) Hepa1.6 cells seeded on glass coverslips were fixed 48 h after transfection with the indicated AAT variants. After permeabilization, cells were immunostained with a polyclonal anti‐human AAT antibody (Dako) (total; green) or with the anti‐polymer 2C1 mAb (polymers; red). Merged panels are shown with overlapping signals in yellow. Nuclei were stained blue by the Hoechst dye. Cells expressing Z and Gly192Cys AAT showed a punctate pattern of 2C1‐positive polymers, which are indicated with white arrows. Scale bar in all panels is 10 μm. The panel is representative of three independent experiments.

To evaluate the basis for the deficiency seen in the proband, Hepa1.6 cells were transiently transfected with Gly192Cys AAT or with the wild‐type M or the Z mutant (Glu342Lys) for reference. The variants were expressed for 20 h and the cells were lysed in 1% v/v NP‐40 detergent in order to resolve the soluble and insoluble intracellular fractions as previously described [16], while cell media was collected to investigate the extracellular secreted fraction. The Gly192Cys mutation introduces a second cysteine in addition to that in the wild‐type protein (Cys232) and so could promote the formation of intra‐ and intermolecular disulphide bonds during folding; this has previously been observed for the I and Brixia variants (Arg39Cys and Phe35Cys, respectively) [17]. The cellular accumulation of Gly192Cys AAT was investigated by resolving both the intracellular and the extracellular components in the presence or absence of reducing agent (DTT). Under non‐reducing conditions, Gly192Cys AAT formed a high molecular weight complex, present in the soluble and insoluble cellular fractions (Fig. 2A, top panel), which was also observed in Z AAT. Additionally, Gly192Cys AAT formed an intrachain disulphide bond (Fig. 2A, marked with an asterisk), which migrated faster than the 52 kDa intracellular monomeric form. This band was present both in the soluble and insoluble fraction and was indeed similar to that seen with the I and Brixia cysteine mutants [17]. These intra‐ and interchain complexes dissociated in the presence of DTT (Fig. 2A, middle panels), confirming they are linked by disulphide bonds.

The accumulation of Gly192Cys AAT in the insoluble fraction suggests that this variant forms intracellular polymers similar to those of Z AAT. To assess this, an immunoprecipitation with the anti‐AAT polymer 2C1 monoclonal antibody [9] was performed (Fig. 2A, bottom panel). The relative levels of the mature glycosylated form in the extracellular fraction indicated a more efficient secretion of Gly192Cys AAT than Z AAT (Fig. 2B). Cellular immunofluorescence showed a characteristic puncta‐like pattern for Gly192Cys AAT, which is consistent with polymers of AAT forming inclusion bodies as observed for the cells expressing Z AAT (Fig. 2C) [9, 18, 19]. This pattern was not seen in cells expressing M AAT, where a majority of the protein signal accumulates in the perinuclear area, consistent with protein processing through the Golgi apparatus. Interestingly, the perinuclear staining pattern was not observed in cells expressing Gly192Cys AAT, which might suggest an increased rate of degradation or a slower rate of protein secretion from the cells.

### The Gly192Cys mutation compromises functional activity

**Fig. 3:**
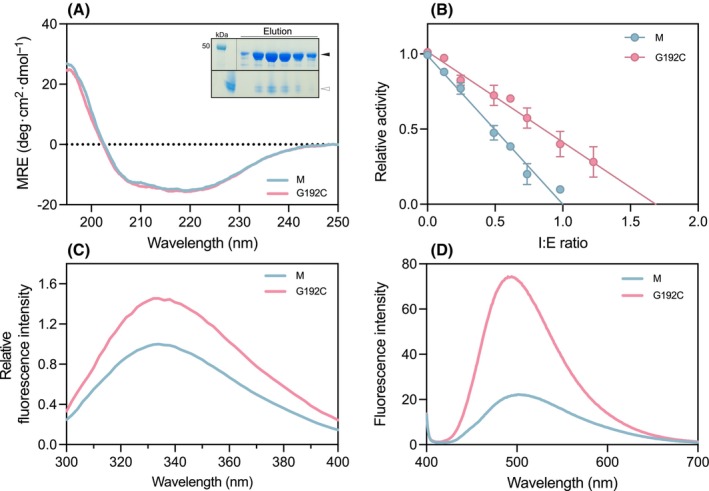
Gly192Cys AAT shows impaired inhibitory activity and polymerisation intermediate‐like properties. (A) Secondary structure assessment by far‐ultraviolet circular dichroism (CD). CD spectra of 0.5 mg·mL^−1^ AAT in 10 mm phosphate buffer were recorded between 195 and 250 nm (*n* = 1). The inset images show purified Gly192Cys AAT fractions eluted following ion‐exchange chromatography separated by 4–12% w/v gradient acrylamide SDS/PAGE (top) and 3–12% w/v gradient acrylamide non‐denaturing‐PAGE (bottom). Arrows indicate bands at the expected molecular weight (black) and in a monomeric state (white). (B) The protease inhibitory activity assay, which measured the residual activity of bovine α‐chymotrypsin at various inhibitor‐to‐enzyme ratios (I : E ratio). The data are the average of three independent experiments (*n* = 3, ± SEM). (C) Intrinsic tryptophan fluorescence spectrum in which 0.3 mg·mL^−1^ of AAT was excited at 270 nm and spectra were recorded between 300 and 400 nm, with the fluorescence intensity normalised to the wild‐type protein. The data represent the average of three technical replicates. (D) Bis‐ANS fluorescence, following incubation of 1 μm AAT variants with 10 μm bis‐ANS for 10 min before recording the spectrum. Samples were excited at 370 nm and spectra were recorded between 400 and 700 nm. The data are the average of three independent experiments. In each graph, M is shown in blue and Gly192Cys (G192C) AAT in pink.

Purified recombinant Gly192Cys AAT (Fig. 3A, *inset*) was subjected to a series of biochemical assays in comparison with recombinant M AAT. The Z variant, which destabilises the breach, leads to an ~ 30% reduction in inhibitory activity [20]. The Gly192Cys mutation had a similar effect with a 1.7‐fold molar excess being required to achieve the complete inhibition of the model protease α‐chymotrypsin (Fig. 3B) [21].

### Fluorescence spectroscopy suggests exposure of a breach region

A series of spectroscopic experiments were used to investigate the bulk solution‐state properties of Gly192Cys AAT in comparison to the wild‐type (M) protein. Far‐ultraviolet circular dichroism spectroscopy (CD) (Fig. 3A) revealed a near‐identical profile for M and Gly192Cys AAT showing that the mutation did not perturb the global structure of the protein. An evaluation of its intrinsic fluorescence profile, which has been shown to primarily report changes in the environment around Trp194 situated within the breach region (Fig. 1), was undertaken. In common with AAT Z [22], the emission profile showed increased fluorescence intensity for Gly192Cys AAT relative to the wild‐type protein (Fig. 3C). However, in contrast to Z AAT, there was no observable shift in the centre of spectral mass (COSM) relative to the wild‐type. This suggests little change of polarity in the immediate environment of Trp194, while the increased fluorescence quantum yield likely reports other rearrangements in the local environment that favour radiative processes over non‐radiative dissipation of energy.

Bis‐ANS is an environmental reporter dye that has been shown to selectively bind Z AAT with respect to the wild‐type protein at moderate temperatures resulting in a pronounced increase in its fluorescence quantum yield [20, 22, 23]. Similarly, in the presence of the Gly192Cys AAT mutant at 25 °C, bis‐ANS exhibited an increased emission intensity (Fig. 3D). In combination, these results suggest similarities in the perturbation of the molecule induced by the novel mutation, with a lesser impact on its global structure.

### Gly192Cys destabilises the intermediate relative to the native state

**Fig. 4:**
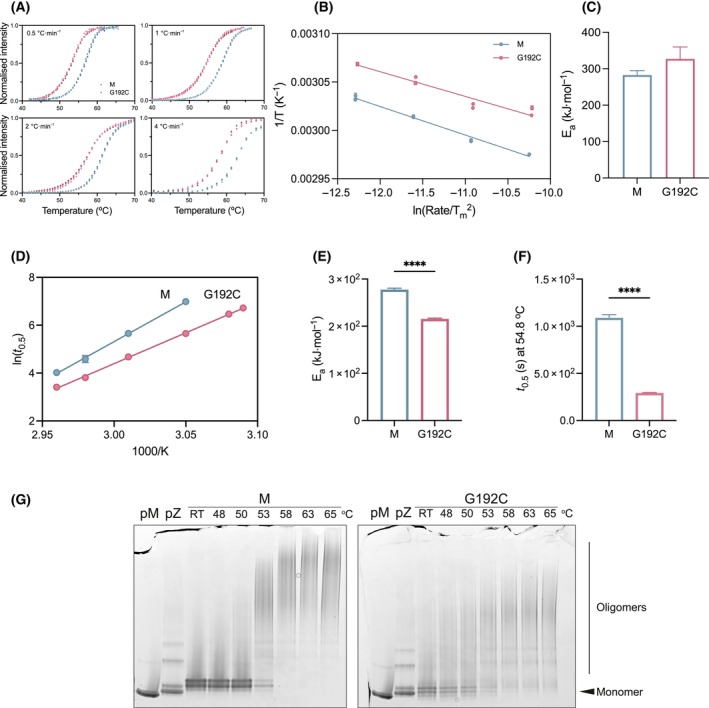
The Gly192Cys mutation destabilises the native and intermediate AAT conformations. (A) The progress curves of thermal shift experiments in which the transition of the monomeric protein was monitored in the presence of SYPRO Orange from 25 to 90 °C at four different rates of temperature increase (°C·min ^−1^ ). (B) The midpoint of the thermal transition (*T*_m_) was plotted as a function of ramp rate, according to the numerical transformations shown. (C) The histograms of calculated apparent activation energy values (*E*_a_) for the transition from native to intermediate states were obtained from the slopes of the regression curves shown in (B). The data are representative of the average of triplicates in two independent experiments, while error bars indicate standard deviation (*n* = 2, ± SD). M and Gly192Cys AAT are shown in blue and pink, respectively. (D) The polymerisation of AAT variants labelled with Atto‐488‐NHS and Atto‐594‐NHS dyes was monitored over a range of temperatures from their increase in relative FRET signal. The polymerisation progress curves were used to determine half‐times of polymerisation (*t*_0.5_). (E) The apparent activation energy for polymerisation (*E*_a_) was calculated from an Arrhenius plot using these values. (F) The half‐time (*t*_0.5_) of polymerisation at a reference temperature (54.8 °C) to provide a typical single‐temperature comparison was interpolated from the Arrhenius plot from the FRET polymerisation assay. (G) A representative non‐denaturing PAGE for the heat‐induced polymerisation assay in which 0.2 mg·mL^−1^ AAT was incubated for 4 h at various temperatures with separation using a 3–12% w/v acrylamide non‐denaturing electrophoresis. Plasma M (pM) and Z (pZ) AAT were used as controls to distinguish the migration pattern of the recombinant proteins. RT represents incubation at room temperature. The statistical significance in (E) and (F) was assessed by unpaired student *t*‐test; **** represents *P* < 0.0001. The data shown in D–F are representative of three separate experiments, error bars indicate standard error mean (*n* = 3, ± SEM).

A thermal stability assay reports the midpoint (*T*_m_) at which 50% of the protein unfolds. In the case of AAT, this ‘unfolded’ state represents the transition to an intermediate ensemble on the polymerisation pathway [24]. The dependence of the *T*_m_ on the rate of heating can allow calculation of the apparent activation energy of the reported transition [25]. M and Gly192Cys AAT were heated from 20 to 94 °C at four rates – 0.5, 1, 2 and 4 °C·min^−1^ (Fig. 4A). At each ramp rate, the observed *T*_m_ for Gly192Cys AAT was lower than that of the wild‐type protein (Table 1). Interestingly, the height of the apparent energy barrier (*E*_a_) for the intermediate state, calculated from the regression slopes of *T*_m_ at different ramp rates (Fig. 4B), was higher for Gly192Cys AAT in comparison with M AAT (326.6 kJ·mol^−1^ SEM ± 33.1 and 282.2 kJ·mol^−1^ ± 12.8, respectively) (Fig. 4C).

**Table 1:** Observed thermal midpoints (*T*_m_) for M and Gly192Cys AAT at four different ramp rates (*n* = 2, ± SD).

Rate	*T*_m_ (°C)			
	0.5 °C·min^−1^	1 °C·min^−1^	2 °C·min^−1^	4 °C·min^−1^
M	56.5 ± 0.3	58.6 ± 0.1	61.4 ± 0.1	63.0 ± 0.1
Gly192Cys	52.7 ± 0.2	54.5 ± 0.4	57.4 ± 0.3	58.0 ± 0.5

### The Gly192Cys mutant leads to an increase in the rate of polymerisation

Polymerisation can be induced artificially under destabilising conditions; thermal stress results in the adoption of a polymer form that shares structural characteristics and exposed epitopes with those that arise naturally in the liver [9, 26]. The kinetics of polymerisation were followed by FRET using AAT labelled with two fluorophores acting as a fluorescence donor and acceptor [20, 21]. The half‐time of FRET increase at temperatures between 50 and 65 °C was represented on an Arrhenius plot as a function of temperature (Fig. 4D). The slopes of the regression lines indicate the energy barrier for polymerisation to progress, which was found to be reduced for the Gly192Cys mutant (Fig. 4E). The shorter half‐times for Gly192Cys AAT are reflected in the shift of the regression line and suggest a faster polymerisation rate. The regression curves also allowed a more accurate comparison, by interpolating from the rates at all temperatures, of the half‐time for polymerisation at a single temperature (Fig. 4F), which at 54.8 °C was 3.8 times faster for Gly192Cys than M AAT.

The susceptibility to polymerisation was also determined by an endpoint assay with visualisation by non‐denaturing‐PAGE (Fig. 4G). Wild‐type and Gly192Cys AAT were incubated for 4 h at a range of temperatures. A higher stability of M AAT was indicated by the presence of the monomeric protein at 48 and 50 °C. Consistent with the results of the thermal stability assay (Fig. 4A, Table 1), the monomeric form was still partially present at 53 °C but completely depleted above 58 °C (Fig. 4G). On the other hand, the Gly192Cys variant started to oligomerise at 48 °C and the monomeric protein was almost completely absent at 53 °C.

### Crystallographic analysis

Crystallisation of Gly192Cys AAT using the hanging drop vapour diffusion method resulted in the formation of plate‐like crystals, which were screened at the Diamond Light Source synchrotron (Harwell Science and Innovation Campus, Didcot, UK). Using the highest resolution dataset, in the C2 space group, the mutant structure was solved at a resolution of 1.9 Å (PDB: 8P4J) (Table 2). A second crystal structure of Gly192Cys mutant (PDB: 8P4U) was also solved at 2.4 Å resolution (Table 2).

**Table 2:** Data collection and refinement statistics^+^.

Data collection	8P4J	8P4U
Wavelength, Å	0.9795	0.9763
Space group	C 1 2 1	C 1 2 1
Cell dimensions		
*a*, *b*, *c* (Å)	114.1, 38.5, 90.0	114.1, 36.4, 88.9
α, β, γ (°)	90, 104.6, 90	90, 106.3, 90
Resolution range (Å)	87.08–1.88 (1.95–1.88)	42.67–2.20 (2.56–2.20)
*I*/δ*I*	1.34	1.5
Completeness spherical (%)	64.9 (12.4)	61.4 (12.1)
Completeness ellipsoidal (%)	84.1 (34.2)	87.5 (50.9)
Refinement		
Resolution (Å)	55.18–1.91 (1.95–1.91)	41.13–2.40 (2.56–2.40)
No. reflections	19 263 (79)	11 027 (298)
*R*_work_/*R*_free_	0.21 (0.31)/0.25 (0.28)	0.21 (0.44)/0.26 (0.51)
No. atoms	2767	2697
*B*‐factors	31.80	50.18
R.m.s. deviations		
Bond lengths (Å)	0.003	0.002
Bond angles (°)	0.560	0.528
Ramachandran plot		
Favoured, %	95.71	95.36
Allowed, %	4.29	4.64
Disallowed, %	0.0	0.0

^+^ Brackets indicate values for the reported high resolution range.

**Fig. 5:**
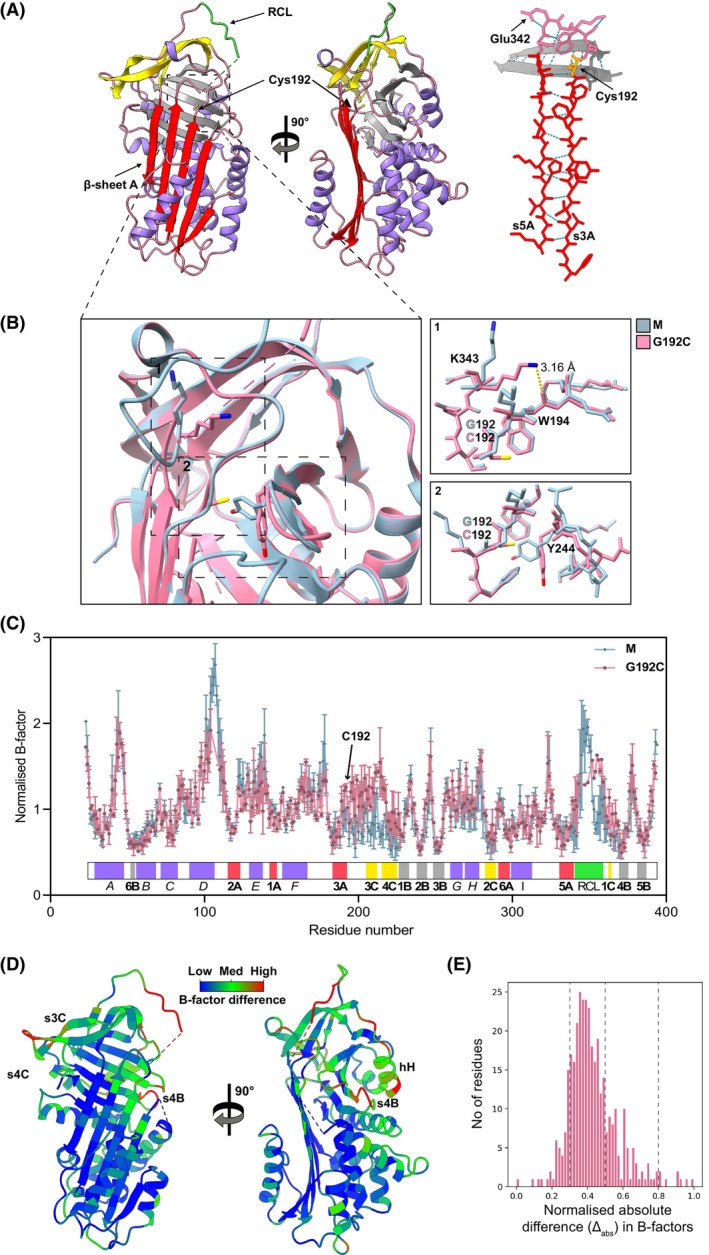
Gly192Cys AAT shows perturbations of side chain packing and dynamics. (A) *Left panel*: The crystal structure of Gly192Cys AAT was solved at 1.9 Å (PDB: 8P4J), shown in the conventional ‘front’ and 90° rotated views. The protein cartoons show α‐helices in purple, β‐sheets A, B and C in red, grey and yellow respectively and the reactive centre loop (RCL) in green. *Right panel*: A coordinate representation of strands 3 and 5 of β‐sheet A (s3A and s5A) in red with Cys192 in orange. The site of the Z mutation (Glu342Lys) is also shown. (B) A comparison of the Gly192Cys mutant protein structure (pink) with wild‐type AAT (M) (PDB: 1QLP, shown in blue). The two regions highlighted in inset panel 1 and inset panel 2, indicate the differences in the orientation of the specific amino acids between the mutant and wild‐type protein. (C) The distribution of the normalised B‐factors in the AAT polypeptide chain, represented by the averages of normalised B‐factor values from two independent structures per variant, M (in blue): 1QLP [27] and 3NE4 [58] and Gly192Cys (in pink): 8P4J and 8P4U. Secondary structure features are represented in a colour bar with indication of α‐helices in italics and β‐strands in bold, respectively. (D) The per‐residue absolute B‐factor differences, obtained by subtracting values for Gly192Cys (PDB: 8P4J) from those of AAT M (PDB: 1QLP), are represented by the blue‐green‐red colour gradient on the Gly192Cys AAT crystal structure. (E) The per‐residue distribution of absolute differences in B‐factors as shown in panel D and normalised between 0 and 1. Representations in panels A and D were generated in chimerax [56], whereas those in panel B were generated in Chimera [59].

In the native structure of Gly192Cys AAT (PDB: 8P4J; Table 2), the additional Cys192, located at the top of strand s3A of β‐sheet A, has a side chain oriented towards the inner face of the protein (Fig. 5A). Interestingly, the cysteine is within the breach, near the disease‐associated Z mutation which is located at the head of strand s5A (Fig. 5A, right panel).

The effect of the mutation was determined by comparison with our crystal structure of M AAT (PDB: 1QLP) [27] based on a backbone alignment of the two molecules (Fig. 5B). The substitution of glycine with cysteine caused structural rearrangements within the breach region. In M AAT, the side chain of Lys343 is normally solvent‐exposed. However, in the Gly192Cys mutant, Lys343 leans towards the loop connecting strand 3A and β‐sheet C, creating a new hydrogen bond with the backbone of Trp194 at a distance of 3.2 Å. Additionally, the presence of the cysteine side chain in the previously unoccupied space (due to the lack of a side chain in glycine) between β‐sheet A and β‐sheet B, resulted in rearrangement of Tyr244. As a consequence, the tyrosine ring is pushed back towards β‐sheet B causing small movements of strands s3B and s4B (Fig. 5C). Tyr244 is conserved in over 70% of serpins [11], and its orientation as found in wild‐type AAT is conserved in over 90 PDB structures of serpins in various conformations deposited in the RSCB Protein Data Bank. This finding makes this mutant structure unique among known mutants of AAT.

Further analysis was undertaken to compare the B‐factors for two M and two Gly192Cys AAT structures (PDB: 1QLP, PDB: 3NE4, PDB: 8P4J and PDB: 8P4U respectively). Increased B‐factor values were evident in the region of the mutation and in the loop connecting strands 3 and 4 of β‐sheet C (Fig. 5C), suggesting higher flexibility or disorder in the mutant. Moreover, slight variations were noticed around α‐helix G and H as well as in the RCL. It is important to note that the RCL is highly flexible due to its protease inhibitory function, as it serves as an exposed docking site for the protease. Consequently, this region is often disordered in AAT structures, and in the case of the structure of Gly192Cys AAT (PDB: 8P4J), eight RCL residues (344–351) lacked sufficient electron density to be modelled.

The differences in normalised B‐factors between Gly192Cys (PDB: 8P4J) and M (PDB: 1QLP) AAT were mapped onto the 8P4J structure to highlight the distribution of differences on the tertiary protein structure (Fig. 5D). The absolute difference in B‐factors revealed the majority of the protein structure to be unchanged or only moderately perturbed. However, more marked differences were observed around the breach region at the site of the mutation along the top of β‐sheet A, suggestive of intrinsic local disorder introduced by Cys192. There was no evidence of a distance‐dependent propagation of increased B‐factors through the molecule (data not shown), which has been observed in NMR chemical shift perturbations in other proteins [28]. The underlying region of strands 4 and 5 of β‐sheet B were also affected which can be explained by the accommodation of Cys192 in the inner cavity within the breach. Interestingly, a B‐factor difference was observed in the solvent‐exposed strands 3 and 4 of β‐sheet C as well as α‐helix H and a loop connecting strands 4 and 5 of β‐sheet B, which are not in direct proximity to the site of the mutation.

### Two conformational forms of Gly192Cys AAT

**Fig. 6:**
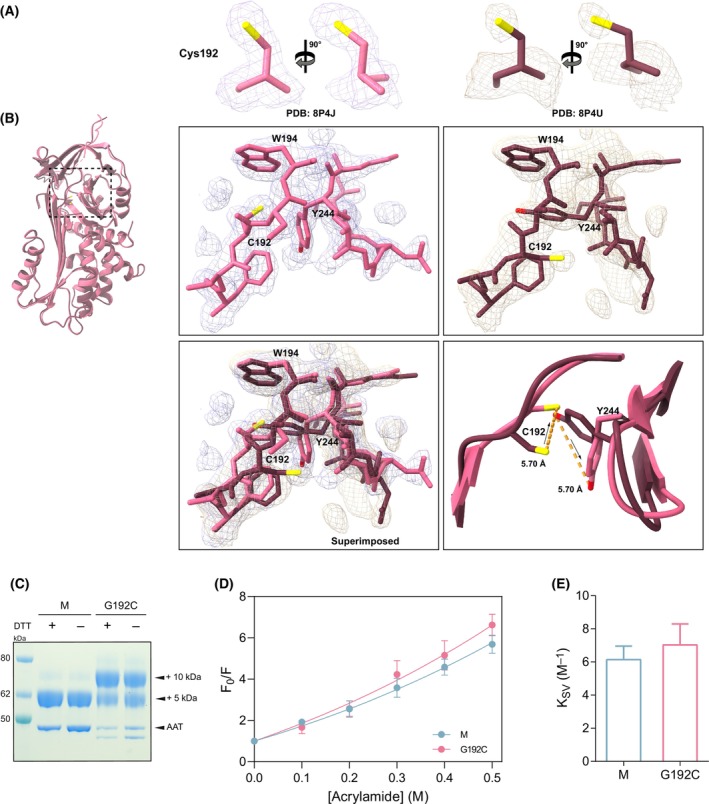
The orientation of cysteine 192 at the mutation site. (A) Placement of Cys192 in the 2*F*_o_‐*F*_c_ electron density maps for two solved structures: 8P4J in pink and 8P4U in dark pink, contoured at 1σ. (B) Structural comparison of the mutation site from two Gly192Cys AAT crystal structures (PDB: 8P4J and PDB: 8P4U). The top panel shows AAT residues 190–194 and 243–249 and their respective 2*F*_o_‐*F*_c_ electron densities, contoured at 1σ. The bottom panels show superimposed structures, with their electron densities (left) and in the ribbon representation with highlighted Cys192 and Tyr244, and the distances between the residues in the two structures (right). (C) A representative 4–12% w/v acrylamide SDS/PAGE gel from the PEGylation experiments (*n* = 3), in which covalent binding of 5k PEG to one cysteine results in a molecular weight shift of 5 kDa. As a control, prior to labelling, protein variants were also reduced with DTT. (D) Stern‐Volmer plots for fluorescent quenching with acrylamide. AAT variants were at 5 μm (blue and pink, respectively for M and Gly192Cys AAT). (E) The quenching constant (*K*_SV_) values, derived from the slope of the regression line in panel (D). The values reported in panels D and E are the averages from three independent experiments, error bars indicate standard deviation (*n* = 3, ± SD).

Only the thiol head of Cys192 could be placed within the electron density of the lower resolution crystallographic dataset (PDB: 8P4U) (Fig. 6A). Electron density was absent from the inner face of the breach region and Tyr244 was shifted back into the breach region pocket as in native AAT, and in contrast to the initial Gly192Cys dataset (PDB: 8P4J). The difference in the shift of Cys192 and Tyr244 between the higher and lower resolution structures was 5.7 Å (Fig. 6B). This suggests that Gly192Cys presents as two conformers, that possibly alternate between one another.

To investigate the apparent rotational flexibility evident at position 192 in the two crystal structures, further biochemical assays were performed to assess the orientation of Cys192. Firstly, M and Gly192Cys AAT were subjected to conjugation with 5 kDa methoxypolyethylene glycol maleimide (5k PEG) which covalently binds free cysteines. AAT has one cysteine at position 232, at the top of strand 1 of β‐sheet B and is accessible for labelling in this way [29], which resulted in a ~ 5 kDa molecular weight shift (Fig. 6C). The conformation of the second cysteine might be determined by either a single or double shift in molecular weight (~ 5 or ~ 10 kDa, respectively). Two labelled populations of the mutant were seen on SDS/PAGE, one with an ~ 5 kDa and one with ~ 10 kDa molecular weight shift (Fig. 6C). This suggests that Gly192Cys AAT is present in two protein conformations, in one of which Cys192 is buried and not accessible for conjugation, and a second in which the mutated residue is solvent exposed.

As described above, it has been found that Trp194 is the primary contributor to the intrinsic fluorescence signal of AAT [30, 31], which sits within the breach region. The effect of the Gly192Cys mutation on the solvent accessibility to Trp194 was assessed in a fluorescent quenching assay in which the tryptophan signal was suppressed by stepwise addition of acrylamide, with the fluorescence intensity plotted as a function of acrylamide concentration (Fig. 6D). The quenching constants (*K*_SV_) derived from the slope of the regression (Fig. 6E) were: 6.1 ± 1.0 M^−1^ (SEM) and 7.3 ± 1.1 M^−1^ (SEM) for M and Gly192Cys AAT, respectively. A higher *K*_SV_ is associated with a faster quenching rate, which indicates increased solvent exposure of the breach region, possibly caused by alternating conformations of cysteine. This may reflect the buried residue reducing quencher access to Trp194 whereas the solvent‐facing cysteine exposes the breach region of the protein. The curvature on the Stern‐Volmer plot was consistent with a ‘quenching sphere of action’ in which proximity of the quencher to some of the fluorophores immediately dissipates excitation energy [32] (Fig. 6D).

## Discussion

**Fig. 7:**
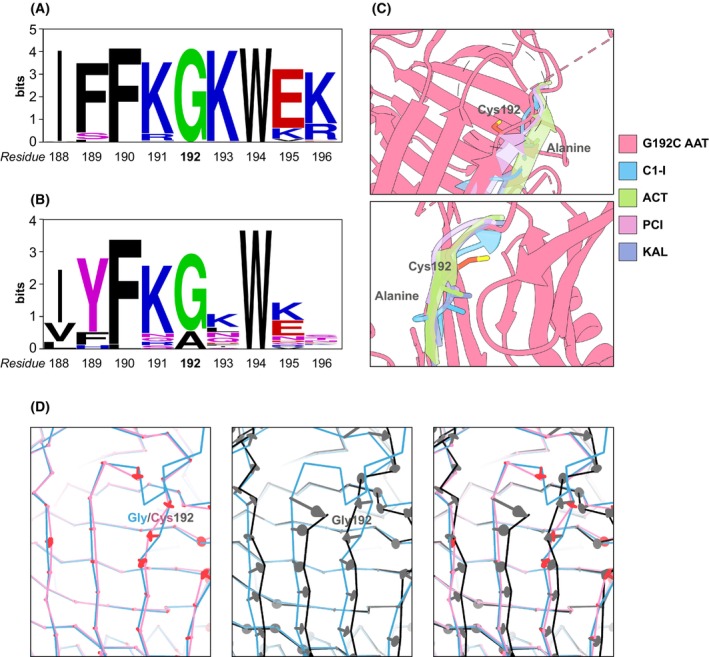
Conservation of glycine 192 in serpins. An alignment of the residues around Gly192 with other AAT proteins across 82 species (A) and with 20 other human inhibitory serpins (B). The conservation analysis was assessed using weblogo online software [54, 55], where Gly192 is coloured green, and other residues are coloured according to their physicochemical properties (black—hydrophobic, blue—basic, red—acidic, magenta—uncharged polar). (C) Breach region in Gly192Cys AAT with superimposed protein fragments of human serpins corresponding to AAT residues 190–194 (*top panel*). Four selected serpins which have alanine in the place of glycine, and their orientations, are indicated in the *bottom panel*. These are C1‐inhibitor (C1‐I, PDB: 5DUQ [60]), alpha‐1‐antichymotrypsin (ACT, PDB: 3DLW [61]), protein C inhibitor (PCI, PDB: 2OL2 [62]) and kallistatin (KAL, PDB: 6F02 [63]), marked by blue, green, pink and purple, respectively. (D) Displacements > 0.5 Å within the backbone of the Gly192Cys AAT crystal structure (PDB: 8P4J, pink) or of a conformation equivalent to the polymerisation intermediate (PDB: 7AEL, black) relative to the native conformation (PDB: 3NE4, blue) are indicated by arrows. Structures in panels C and D were generated in chimerax [56].

The novel Gly192Cys mutation was identified in an individual with low circulating levels of AAT. The site of the substitution is in the breach region which is involved with both inhibitory activity and polymerisation. Formation of the AAT:protease complex requires a rapid conformational transition which results in the opening of β‐sheet A and insertion of the RCL as strand 4A [33, 34] initiated within the breach region [13]. The importance of this for the serpin superfamily is reflected in the generally high sequence conservation within the breach [11]. Wild‐type AAT sequence alignments with human inhibitory serpins and sequences of AAT from different species support a highly conserved glycine at position 192 (Fig. 7A,B). Among amino acids, glycine exhibits the greatest degree of rotational freedom due to the lack of a side chain. Conservation of this residue at the top of strand 3A therefore suggests a requirement for torsional flexibility for the proper functioning of the breach, as the protein goes through a series of intermediate states to achieve the thermodynamically favourable loop‐inserted form [35]. Consistently, we observed an ~ 40% decrease in inhibitory activity of the AAT Gly192Cys variant, whose cysteine residue imposes a greater torsional restriction (Fig. 3B). In a comparison of 21 inhibitory human serpins, most also had a glycine residue, although four instead tolerated an alanine at this position. A superimposition of crystal structures indicated that these serpins have adapted to this residue by adopting a common side chain orientation away from the breach, which differs from that seen in the variant structures reported here (Fig. 7C).

These crystallographic studies identified two orientations of Cys192 within Gly192Cys AAT (Fig. 6). In the 8P4J structure, Cys192 faces towards the inner face of the protein, leading to notable rearrangements, especially of the highly conserved Tyr244 residue. Interestingly, in the 8P4U structure, where the cysteine is solvent‐exposed, Tyr244 is repositioned to the canonical orientation (Fig. 6). The presence of a mixed monomeric population in solution was confirmed by the PEGylation assay with a visible double shift in molecular weight by the conjugation of both single and double adducts (Fig. 6C), and supported by the acrylamide quenching assay which revealed differential accessibility to Trp194 within the breach region (Fig. 6D). The crystal structures explain the effect of the mutation with opening within the breach region and movement of β‐sheet B and helix H (Fig. 5).

In the process of thermal ‘unfolding’, AAT is induced to form a polymerisation‐prone intermediate (M*), which then irreversibly associates with another activated molecule to form polymers (*P*) [36] as shown in Eqn (1).

(1) M⇄M*+M*→P

The thermal denaturation experiments allowed derivation of the apparent activation energy (*E*_a_) of this change [25]. Paradoxically, while the native configuration was destabilised, the relative *E*_a_ barrier for formation of the intermediate was higher for Gly192Cys in comparison with M AAT (Fig. 4B,C). The structure of the polymerisation intermediate can be inferred from a small molecule‐bound form of AAT [37, 38]; interestingly, in this form the backbone is perturbed in the vicinity of position 192, relative to the native state (Fig. 7D). Interchange between the buried and exposed orientations of the cysteine would be reduced in comparison with the torsional freedom of a glycine residue, partially impeding intermediate formation, but favouring polymer formation over reversion to the native conformation once it has formed. Indeed, the thermal stability experiments indicated a lower degree of reversibility to the native state.

A further possible contributor to the increased activation barrier is the observed structural perturbation of Lys343 (Fig. 5B, inset panel 1) which involves the formation of a new stabilising hydrogen bond. Further biochemical analysis indicated a difference in the interaction with the environmental reporter bis‐ANS [22], which has been found to recognise an intermediate conformation of ΑΑΤ that is populated by the Z variant of AAT and by wild‐type M ΑΑΤ when it is heated [39]. Similar to Z AAT, Gly192Cys AAT induced a much higher fluorescence intensity in the dye over the wild‐type protein (Fig. 3D). While the site of interaction of this molecule has not been established, the observed increased movements of hH and s3B and s4B suggests that destabilisation of β‐sheet B (Fig. 5D) may be involved in this process.

Once activated into the polymerisation‐prone intermediate, the energy barrier for the irreversible self‐association of the mutant with another molecule is significantly reduced, leading to a faster rate of polymerisation upon thermal treatment in comparison to the wild‐type protein (Fig. 4). When expressed in Hepa1.6 cells, Gly192Cys AAT shared with Z AAT the ability to form intracellular polymers and inclusion bodies within the ER of cells (Fig. 2). This accumulation has also been observed in other rare variants such the highly polymerogenic Baghdad (Ala336Pro) [20] and King's (His334Asp) [9] AAT in which the functional opening of β‐sheet A is impaired. It is also possible that Gly192Cys and Z AAT may interact and co‐polymerise during expression, by analogy with other variants [40, 41].

The impact of the mutation on the severity of the liver and lung disease is hard to characterise clinically as the index case is also a carrier of the severely defective Z allele. Nevertheless, experiments performed in a mammalian expression system, as well as biochemical, biophysical and crystallographic studies, collectively demonstrate that the Gly192Cys mutation promotes formation of AAT polymers associated with an increased risk of liver disease, and depletion of functional levels associated with lung disease. However, this is likely to be to a lesser degree than that seen with homozygosity for the severe Z deficiency allele.

## Materials and methods

Unless otherwise noted reagents were obtained from Sigma Aldrich (St. Louis, MO, USA)/Merck (Darmstadt, Germany).

### Cloning and plasmid preparation

The QuikChange II site‐directed mutagenesis kit (Agilent, Santa Clara, CA, USA) was used to introduce the single point mutation (Gly192Cys) into wild‐type AAT encoded by recombinant and mammalian vectors. Recombinant proteins were expressed using the pQE‐30 vector (Qiagen, Hilden, Germany) containing the AAT sequence with an N‐terminal hexa‐histidine tag for affinity chromatography [24]. The expression vectors used for the mammalian cell culture assays were as previously reported [42] and based on those encoding human M1 (Val213) and Z AAT.

### Recombinant protein expression and purification

Recombinant AAT was expressed in XL1‐Blue *E. coli* cells (Agilent) and purified by affinity chromatography as described previously [24] with the addition of 100 mm β‐mercaptoethanol prior to ion‐exchange. The yield of monomeric Gly192Cys AAT was ~ 8 mg·L^−1^ of culture, approximately 50% lower than that of M AAT under equivalent conditions. Protein homogeneity was established by non‐denaturing and SDS/PAGE electrophoresis. The resulting purified protein was buffer‐exchanged into either PBS (PBS pH 7.4, 0.02% w/v NaN_3_) or crystallography buffer (10 mm Tris pH 7.4, 50 mm NaCl, 0.02% w/v NaN_3_) and stored at −80 °C.

### Mammalian cell culture

The Hepa1.6 mouse hepatoma cell line (ATCC CRL‐1830) was grown in Dulbecco's modified eagle medium (DMEM) (Sigma Aldrich) supplemented with 10% v/v foetal bovine serum. Transient transfections were performed with polyethyleneimine ‘MAX’ (PEI) (Polysciences, Warrington, PA, USA) as previously described [17]. Transfected cells were then washed twice with pre‐warmed PBS and further incubated at 37 °C with 1 mL of Opti‐MEM (Gibco, Thermo Fisher Scientific, Waltham, MA, USA). After 20 h incubation, cell culture media were collected, centrifuged at 800 ***g*** for 5 min at 4 °C, transferred into clean tubes and stored at −20 °C, while the cells were lysed as previously described [16].

Hepa 1.6 cells, extensively utilised for AAT studies [16, 43, 44, 45] had been procured from and authenticated by Merck KGaA (Darmstadt, Germany) < 3 years before the first transfection. All experiments were conducted using cells routinely screened for mycoplasma contamination.

### Cellular immunofluorescence

As previously described in Ronzoni *et al*. [44], Hepa 1.6 cells were seeded onto 2 cm^2^ coverslips (Millipore Sigma, Burlington, MA, USA) and transfected as described above. After 48 h, the cells were fixed with 4% v/v paraformaldehyde, permeabilised with 0.1% v/v Triton X‐100, and immunodecorated with anti‐human AAT (Agilent Dako, Glostrup, Denmark) (at a concentration of 2.2 μg·mL^−1^) or the anti‐AAT polymer 2C1 mAb [9] (0.8 μg·mL^−1^) overnight at 4 °C. The primary antibodies were detected with goat anti‐mouse antibody conjugated to Alexa Fluor 488 and goat anti‐rabbit antibody conjugated to Alexa Fluor 555 (Thermo Fisher Scientific). Cells were counterstained with Hoechst (Thermo Fisher Scientific) to visualise the nuclei. Coverslips were mounted on slides with Immuno‐Mount (Thermo Fisher Scientific) and analysed on a Zeiss Airscan 880 confocal microscope with a 63× objective (1.4 oil).

### Biochemical assays

The stoichiometry of inhibition (SI) of AAT against α‐chymotrypsin was determined as described previously [21]. Intrinsic fluorescence spectra were recorded on a SpectraMax M5 plate reader (Molecular Devices, San Jose, CA, USA), whereas the bis‐ANS readout was undertaken using a Cary Eclipse fluorescence spectrophotometer (Agilent Technologies), both at 25 °C [20]. For intrinsic fluorescence, samples were excited at 270 nm and scanned in the emission range from 290 to 450 nm. Bis‐ANS was incubated with AAT for 10 min at room temperature prior to recording an emission scan between 400 and 700 nm with 10 nm slits for both emission and excitation and an excitation wavelength of 370 nm. Circular dichroism (CD) spectra were recorded at 25 °C on J‐720 spectropolarimeter (Jasco, Inc., Easton, MD, USA) in the ISMB Protein Crystallography and Biophysics Centre (BiophysX). Protein samples were prepared in a low ionic strength buffer as described [21]. The stability of the AAT variants was determined in a SYPRO Orange‐based thermal denaturation assay as described previously [20, 21] (Thermo Fisher Scientific). The midpoint of denaturation (*T*_m_) was determined by fitting the data to a nonlinear regression algorithm describing a two‐state unfolding transition [24]. The kinetics of polymerisation for AAT labelled with Atto‐488‐NHS and Atto‐594‐NHS (Atto‐Tec, Siegen, Germany) via primary amines were assessed in a FRET‐based assay [20, 21, 24] using a RealPlex4 MasterCycler qPCR instrument (Eppendorf, Hamburg, Germany). Polymerisation was determined by an increase in FRET recorded at an emission wavelength 605 ± 15 nm upon excitation at 470 nm over a range of temperatures.

### Crystallography and structure determination

Crystals of Gly192Cys AAT were grown in 100 mm of MIB buffer (sodium malonate dibasic monohydrate, imidazole, boric acid) (Molecular Dimensions, Sheffield, UK) and 25% v/v 1500 polyethylene glycol (Sigma). The protein used for crystallography was buffer‐exchanged into crystallography buffer (10 mm Tris pH 7.4, 50 mm NaCl, 0.02% NaN_3_) and concentrated to 11 mg·mL^−1^. Crystals were grown by the hanging drop technique when mixed in a 1 : 1 ratio with the growth buffer.

Fully‐formed crystals (usually 7–10 days post‐seeding) were snap‐frozen in liquid nitrogen in the presence of cryoprotectant. Data collection was undertaken at the Diamond Light Source on the I03 beamline (Harwell Science and Innovation Campus). The diffraction data were recorded over 360° with 0.2° oscillation/frame. Collection conditions were as follows: beam wavelength 0.9763 Å, beam size 80 × 20 μm and average photon flux 1.95 × 10^12^ and the exposure time was 0.05 s.

The collected diffraction data were autoprocessed with autoPROC and STARANISO [46, 47] which performs an elliptical truncation of data and relies on other software, including XDS for space group determination and integration of diffraction data [48] and Aimless for merging and scaling [49].

The crystal structures were solved by molecular replacement with the native AAT structure [27] (PDB: 1QLP) used as an initial search model in Phaser [50], the atomic protein models were refined using phenix [51], and model building was undertaken with the coot software [52]. The final crystal structures were validated using the online wwPDB validation server [53].

### Polyethylene glycol conjugation assay

Conjugation of 5 kDa methoxypolyethylene glycol maleimide (5k PEG) (Sigma Aldrich) was undertaken by incubating the AAT variants at 0.5 mg·mL^−1^ (11.1 μm) with a 10‐fold molar excess of 5k PEG for 30 min at room temperature [29]. The PEGyaltion reaction was stopped by quenching with free L‐cysteine at a 10‐fold molar excess relative to 5k PEG for 1 h at room temperature. The verification of successful conjugation was by molecular weight shift on SDS/PAGE.

### Acrylamide‐induced protein quenching assay

The quenching of tryptophan fluorescence was induced by acrylamide as recorded on a Cary Eclipse fluorescence spectrophotometer (Agilent Technologies). The wavelengths for excitation and emission were 280 and 340 nm, respectively. Five micromolar of AAT was prepared in a total volume of 90.5 μL and 0.1 m of acrylamide was added five times stepwise upon the stabilisation of the fluorescence signal, to a final reaction volume of 100 μL.

The average of the raw fluorescent signal at each concentration of acrylamide (*F*) was normalised to the protein signal without acrylamide (*F*_0_) and corrected for the changing reaction volume. The normalised fluorescent signal was plotted as a function of the acrylamide concentration. Data were plotted using a correction factor for the ‘quenching sphere of action’ effect [32] as follows:

$$F_{0}/F=\left(Y_{\mathrm{int}}+K_{\mathrm{SV}}\left[Q\right]\right)*e^{\left(V\left[Q\right]\right)}$$

where *K*_SV_ is the Stern‐Volmer quenching constant, [*Q*] is the quencher concentration, *Y*_int_ is the intercept of the line (around 1) and *V* is related to a volume in which nearby quenchers entirely suppress fluorescence.

### Sequence analysis

WebLogo [54, 55] was used to analyse and represent patterns of conservation. Sequences of antitrypsin orthologues were obtained from UniProt with the following accessions: P01009, A0A2Y9FBB2, A0A673U4E0, A0A096NJJ1, A0A5F8ANV5, A0A4X2KQL0, M3XVV7, A0A2Y9JIB0, Q00896, A0A5N3X4Z1, F1SCF0, A0A1U8BK25, A0A452RLL8, A0A383ZV20, O54763, H0WJ03, G3QXZ8, W5PZS7, A0A5F4WK65, A0A7J7RA69, L5JP19, Q64118, A0A6I9M7A9, A0A6P6EST7, A0A2K5P2E8, A0A341CI16, A0A6P3RA28, A0A2Y9NAM4, F7DXM5, G3I296, A0A0D9REV5, A0A6J2L6R7, A0A3Q7QYY0, Q63969, G5B496, A0A2U3VIL9, P26595, A0A3P4MYE3, M3WCX1, Q03044, A0A091CQE0, A0A671EGC0, L5LNJ6, A0A6P5LTY0, A0A6P6HF76, A0A7J8IT29, A0A2K5ID60, A0A2Y9DLF9, A0A2J8QMJ1, A0A6J0XZP9, A0A1A6GDH5, A0A1S3A4F6, L8YAZ3, A0A6I9J6U5, A0A2K5R2K3, A0A340WJU5, D2HEM3, G3TDW3, G7PBD8, A0A1S3FA35, A0A0G2JZ73, A0A1U7SNR2, A0A5E4D9W6, A0A2K5XHH4, A0A212CS37, A0A2K5DME6, A0A6J3ILL6, A0A2K6TI38, A0A2K6MV27, A0A6I9ZXC6, A0A6J3QVN9, F1PCE5, A0A6P3H5V3, G1S644, A0A452FJ07, O54761, Q5RCW5, A0A7J7QTL4, O54757, P22325, A0A485MCA2, and P23035. Sequences of other inhibitory serpins were from UniProt with accessions: P01009, P01011, P05154, P29622, I38201, I38202, P35237, P50452, P50453, P48595, P05120, P30740, BAA31232, P01008, P05546, P05121, P07093, P08697, P05155, Q99574, O75830.

## Conflict of interest

The authors declare no conflict of interest.

## Author contributions

KK and RR performed experiments. JAI, KK, RR and DAL analysed the data and wrote the manuscript. AM and FHXG identified the individual with the mutation and undertook clinical measurements. JAI and DAL supervised the work. All authors read, revised, and approved the final manuscript.

### Peer review

The peer review history for this article is available at https://www.webofscience.com/api/gateway/wos/peer‐review/10.1111/febs.17121.

## Data Availability

The atomic coordinates and structure factors for the Gly192Cys AAT structures have been deposited in the RCSB Protein Data Bank with accession codes 8P4J and 8P4U, with other published structures and sequences obtainable from the RCSB Protein Data Bank and UniProt databases, respectively.
